# Reply to: Limitations of molecular testing in combination with computerized tomographic for lung cancer screening

**DOI:** 10.1038/s41467-022-31420-2

**Published:** 2022-07-08

**Authors:** Dimitrios Mathios, Peter B. Bach, Jillian A. Phallen, Robert B. Scharpf, Victor E. Velculescu

**Affiliations:** 1 The Sidney Kimmel Comprehensive Cancer Center, Johns Hopkins University School of Medicine, Baltimore, MD USA; 2 Department of Neurosurgery, Johns Hopkins University School of Medicine, Baltimore, MD USA; 3 Delfi Diagnostics, Baltimore, MD USA; 4 Department of Biostatistics, Johns Hopkins Bloomberg School of Public Health, Baltimore, MD USA

**Keywords:** Cancer genomics, Diagnostic markers

**replying to** Grannis Jr. et al. *Nature Communications* 10.1038/s41467-022-31419-9 (2022)

In the Mathios et al. study^1^, we demonstrated that a fragment-based cell-free DNA classifier (DELFI) evaluated on the prospectively collected participants in the LUCAS cohort could distinguish between patients with and without lung cancer and that the performance extended to an external validation dataset. We also demonstrated that DELFI could delineate patients with small cell lung cancer from other lung cancers and that the overall score was independently predictive of patient prognosis after adjustment for clinical factors including age, cancer stage, histological subtype, and treatment (Supplementary Fig. 11 in previous study).

In his comment on our study, Dr. Grannis raised the concern that our analytic cohort included many individuals with symptoms suggestive of lung cancer, rather than a symptom-free screening cohort. We highlighted this divergence in our paper too, but several points might alleviate this concern. First, we note it is common to initially evaluate cancer screening modalities in symptomatic individuals. The mammogram, colonoscopy, and prostate specific antigen test were all originally used for the evaluation of symptoms and signs of disease^2–4^. Additionally, according to the United States Preventative Task Force (USPTF) recommendation, individuals eligible for computed tomography (CT) screening include those with significant smoking history. The majority of our patients who were symptomatic had dyspnea and cough, symptoms common in the eligible population of heavy smokers that would be included in a screening study. We also included individuals with a prior history of cancer and comorbidities such as autoimmune and inflammatory conditions, traits that would be present in real-world screening populations.

We agree with Dr. Grannis regarding the proper sequence of steps for the development of an early detection test and re-emphasize that our analyses were proof of concept, building on another set of fragment based analyses we previously described across multiple cancer types^5^. Further development of a DELFI classifier using screening cohorts is still needed and Dr. Grannis points to a study that has already been launched: the L101 study aims “to train and test classifiers for lung cancer detection using the DELFI assay”, and focuses on individuals eligible for CT screening^6^.

Dr. Grannis expresses curiosity regarding the size of nodules in our study, based on his theory that the size of the nodule is itself a predictor of the clinical benefit of detecting it. Our study captured nodule size using the widely recognized TNM staging system and more detailed nodule dimensional measurements were not available for the cohorts we analyzed. Future analyses with cohorts with detailed radiographic annotation of lung nodules would be of value to assess the performance of the DELFI approach in detecting very small lung cancer lesions.

Dr. Grannis notes that a biomarker assay could harm patients by missing some sub-centimeter lung cancers. We agree that any biomarker must be assessed for its ability to identify patients with the earliest stage of disease, and that high false negative rates for small lesions would be a serious concern. However, the importance of the false negative rate of any biomarker for lung cancer should be balanced with the current low uptake of CT screening. A study by Jemal et al.^7^ cited a rate of 3.3–3.9% of eligible individuals undergoing CT imaging. A blood based assay could ameliorate this problem if two somewhat safe assumptions are met: a blood test would be adopted more easily by physicians as a first assessment for lung cancer than CT screening has been and, following a positive test, a CT scan would be ordered with high frequency.

The potential harms of CT screening include the frequency of false positives, the anxiety and cost associated with the test, and the high levels of exposure to ionizing radiation even at low doses. The benefits derive almost exclusively from the identification of patients with undiagnosed lung cancers who can then receive treatment. A blood-based biomarker could favorably shift the risk-benefit tradeoff of CT screening by identifying the subset of eligible individuals who are most likely to have lung cancer. Each positive CT scan would be more likely to identify a treatable cancer (higher positive predictive value), favorably shifting the true positive to false positive ratio and sparing many of those who are unlikely to have lung cancer from the harms associated with CT scanning.

This potential use of blood based early detection as a means of minimizing risks associated with CT screening is in line with the improvements in lung scan interpretation we have witnessed over the past decade. The Lung-RADS algorithm for CT scan evaluation and management, when applied to the NLST cohort retrospectively, reduces the sensitivity for lung cancer from 93.5% to 84.9%, but provides more than a 50% reduction in the baseline false positive rate (26.6–12.8%)^8^. I-ELCAP investigators, also in an analysis of NLST data, concluded that “higher thresholds of nodule size should be considered and prospectively evaluated”^9^. At the extremes the authors contemplate the impact of increasing the threshold for nodule evaluation from 5 mm to 9 mm. The higher threshold for nodule evaluation would more than halve the false positive rate (10.5–4.1%) but lead to delays in lung cancer diagnosis of greater than 9 months for 9.9% of patients by reducing the sensitivity of CT screening.

In conclusion, Mathios et al. demonstrate that non-invasive lung cancer detection is feasible through evaluation of genome-wide cfDNA fragmentation. Two larger trials, focused on CT-eligible screening populations are ongoing (NCT04825834, DELFI-L101 and NCT05306288, CASCADE-LUNG) with the goal of training and validating a classifier for early detection of lung cancer. Given the low depth of whole genome sequencing utilized by the DELFI approach, a test based on this method could provide an affordable and highly accessible avenue for population-scale lung cancer screening.
